# Novel endoscopic full-thickness resection device for colon polyp resection

**DOI:** 10.1055/a-2563-1474

**Published:** 2025-04-09

**Authors:** Tessa Herman, Rahul Karna, Roberto Osorio-Cintron, Natalie Wilson, Daniela Guerrero Vinsard, Brian J Hanson, Mohammad Bilal

**Affiliations:** 1 14400 Department of Medicine, University of Minnesota Medical Center, Minneapolis, United States; 2 14400 Division of Gastroenterology, Hepatology, and Nutrition, University of Minnesota Medical Center, Minneapolis, United States; 3 Gastroenterology Section, Minneapolis Veteran Affairs Medical Center, Minneapolis, United States; 4 129263 Division of Gastroenterology and Hepatology, University of Colorado Anschutz Medical Campus, Aurora, United States

Endoscopic full-thickness resection (EFTR) allows for the management of scarred and fibrotic lesions that are not amenable to endoscopic mucosal resection or endoscopic submucosal dissection (ESD). Two main methods of performing EFTR include exposed and nonexposed EFTR. In exposed EFTR, the lesion is resected followed by defect closure. In nonexposed EFTR, the defect is closed prior to resection of the lesion.

**Fig. 1:**
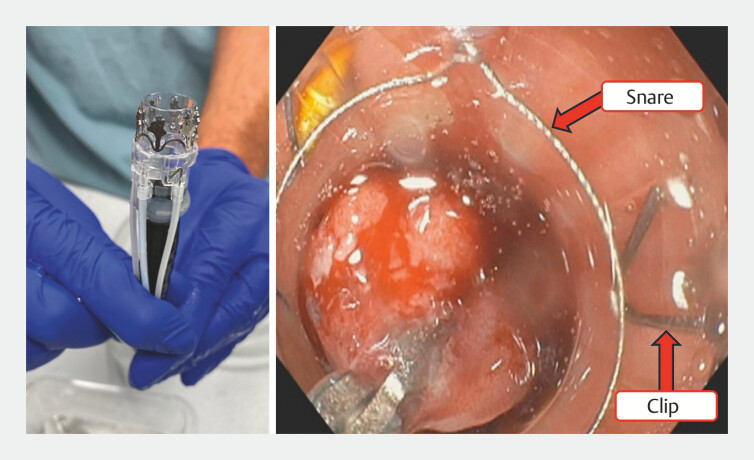
Dedicated, novel full-thickness resection device that includes a clip and pre-loaded snare for lesion resection.

The Padlock FTR System (Steris, Mentor, Ohio, USA) is a novel full-thickness resection (FTR) device that includes a Padlock design clip to achieve nonexposed EFTR and then uses a pre-loaded snare to resect the lesion ( Fig. 1 ). To the best of our knowledge, we show the first reported case of in-human use of this device ( Video 1 ).

Resection of a large sigmoid polyp using a novel full-thickness resection device.Video 1

**Fig. 2:**
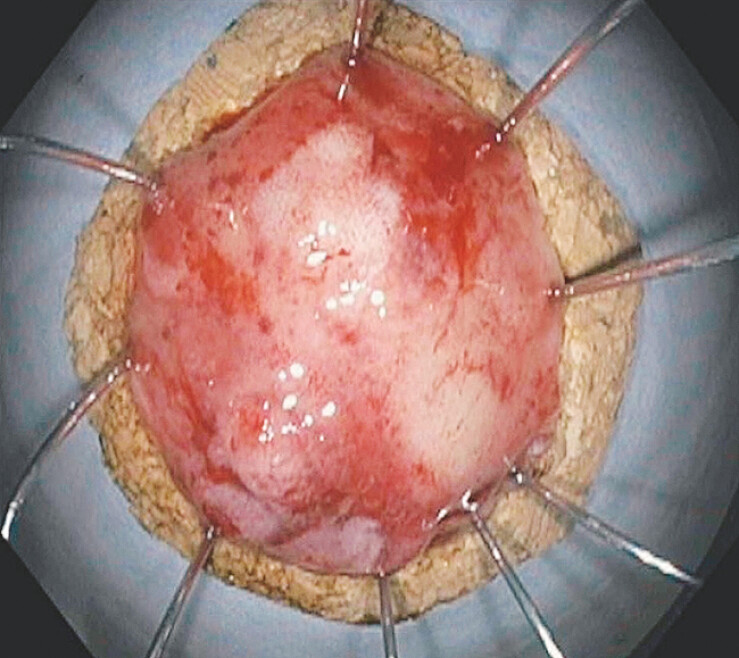
Successful resection of the 20-mm sigmoid polyp using the novel full-thickness resection device.

**Fig. 3:**
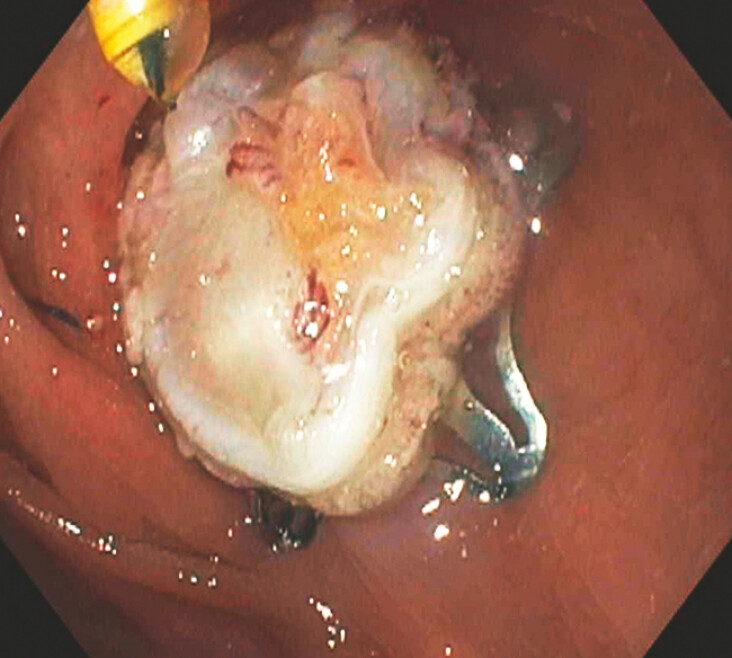
Post-resection examination showed the fatty patch, consistent with full-thickness resection. The clip was in the appropriate position.

**Fig. 4:**
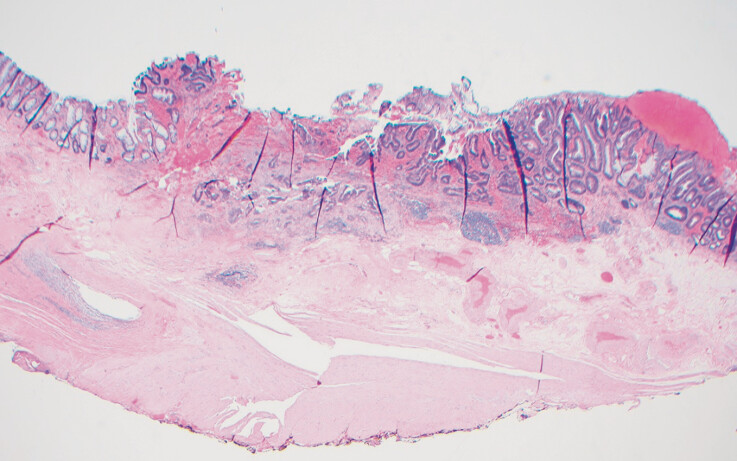
Invasive adenocarcinoma with submucosal invasion to a depth of 600 microns. Deep and lateral margins were negative (R0 resection).

A 60-year-old man with a history of >20 polyps and prior ESD for three 4- to 6-cm rectosigmoid polyps presented for endoscopic resection of a rectosigmoid polyp adjacent to a prior ESD resection site. A 20-mm sessile polyp was found in the sigmoid colon next to a previously placed cinch at the site of a prior polyp resection. Given extensive scarring at the site, the decision was made to pursue EFTR. The polyp was successfully resected using this novel FTR device ( Fig. 2 ). No adverse events occurred. Examination of the resection site showed the fatty patch, consistent with full-thickness resection, and the clip in place ( Fig. 3 ). Pathology revealed a well-differentiated, invasive adenocarcinoma with a depth of submucosal invasion of 600 microns ( Fig. 4 ). Deep and lateral margins were negative (R0 resection) for dysplasia.

This device could be a useful tool in the armamentarium for EFTR of previously manipulated colon polyps and lesions with superficial submucosal invasion. Further reports are needed to confirm its safety and efficacy.

Endoscopy_UCTN_Code_TTT_1AQ_2AD_3AF

